# Safety and efficacy of Tolvaptan in real-world patients with autosomal dominant polycystic kidney disease- interim results of SLOW-PKD surveillance

**DOI:** 10.1007/s10157-021-02100-0

**Published:** 2021-07-06

**Authors:** Toshio Mochizuki, Satoru Muto, Masateru Miyake, Toshiki Tanaka, Wenchyi Wang

**Affiliations:** 1 Department of Nephrology, Tokyo Women’s Medical University, Tokyo, Japan; 2 Clinical Research Division for Polycystic Kidney Disease, Department of Nephrology, Tokyo Women’s Medical University, Tokyo, Japan; 3 Department of Urology, Graduate School of Medicine, Juntendo University, Tokyo, Japan; 4 Department of Advanced Informatics for Genetic Disease, Graduate School of Medicine, Juntendo University, Tokyo, Japan; 5 Department of Pharmacovigilance, Otsuka Pharmaceutical Co., Ltd, 3-2-27 Otedori, Chuo-ku, Osaka, 540-0021 Japan; 6 Department of Medical Affairs, Otsuka Pharmaceutical Co., Ltd, 3-2-27 Otedori, Chuo-ku, Osaka, 540-0021 Japan; 7 Otsuka Pharmaceutical Development & Commercialization, Inc, Princeton, NJ USA

**Keywords:** Autosomal dominant polycystic kidney disease, Post-marketing survey, Glomerular filtration rate, Total kidney volume, Safety profile, Tolvaptan

## Abstract

**Background:**

Tolvaptan is a vasopressin type 2 receptor antagonist and has been used to treat autosomal dominant polycystic kidney disease (ADPKD) since 2014. There has been limited real-world data on the safety and efficacy of tolvaptan.

**Methods:**

This post-marketing surveillance was conducted to evaluate the long-term safety and the efficacy of tolvaptan in Japanese patients with ADPKD in real-world clinical settings. The baseline characteristics of 1630 patients treated with tolvaptan are reported. Safety analysis comprises evaluation of adverse drug reactions (ADRs). The efficacy evaluation includes percent change in total kidney volume (TKV) and change in estimated glomerular filtration rate (eGFR) before and after tolvaptan treatment.

**Results:**

Mean age was 49.7 ± 11.2 years and 843 (51.7%) patients were male. Baseline TKV was 2158 ± 1346 mL and eGFR was 44.4 ± 21.7 mL/min/1.73 m^2^. The majority of CKD patients were stage G3b (27.0%) and G4 (30.1%). Frequently reported ADRs were hepatic function abnormal (8.3%), thirst (8.2%), and hyperuricaemia (6.9%). The frequency of ALT elevation (> 30 and > 90 IU/L) was slightly high (32.9 and 8.3%) to previous studies. After tolvaptan treatment, the annual rate of percentage change in TKV reduced from 11.68%/year to 2.73%/year (*P* < 0.0001). Similar results were also obtained for the effect on change in eGFR from − 3.31 to − 2.28 mL/min/1.73 m^2^/year after initiation of tolvaptan treatment (*P* = 0.0403).

**Conclusion:**

There were no major problems with safety of tolvaptan treatment and comparable efficacy for TKV and eGFR was observed in relation to the previous pivotal two randomized control trials in this post-marketing surveillance.

**Supplementary Information:**

The online version contains supplementary material available at 10.1007/s10157-021-02100-0.

## Introduction

Autosomal dominant polycystic kidney disease (ADPKD) is the most common hereditary monogenic kidney disorder and the fourth leading cause of end-stage kidney disease (ESKD) in adults worldwide [1, 2]. It is a heterogeneous disorder resulting from mutations in two genes, *PKD 1* and *PKD 2* [1, 2]. ADPKD occurs in all races worldwide and its prevalence is estimated to be 1/4000 in Japan [3], and less than 5 in 10,000 in Europe according to the most recent data [4]. The clinical hallmark of ADPKD is the development of fluid-filled renal cysts leading to organ enlargement, chronic kidney disease (CKD), and extra-renal complications such as hypertension, liver cyst and intracranial aneurysm [5–7].

Mutations of the *PKD 1* or *2* gene result in impaired calcium entry into renal tubular epithelial cells. Low intracellular calcium triggers increased adenosine-3’, 5’-cyclic monophosphate (cAMP) levels. This plays a major role in cyst development and contributes to the development of ADPKD. The arginine vasopressin (AVP) independently upregulates cAMP, which further increases cell proliferation and fluid secretion [8]. Tolvaptan, an oral selective vasopressin V_2_ receptor antagonist, decreases fluid secretion and cell proliferation, thus slowing disease progression [9, 10].

The phase 3 clinical trial, Tolvaptan Efficacy and Safety in Management of Autosomal Dominant Polycystic Kidney Disease and Its Outcomes (TEMPO 3:4), was conducted to evaluate the efficacy of tolvaptan treatment over 3 years [11]. The study included 1445 patients with ADPKD with a total kidney volume (TKV) of ≥ 750 mL and an estimated creatinine clearance (CCr) ≥ 60 mL/min. The results showed a reduced annual rate of percentage change in TKV and a slower annual rate of kidney function decline with tolvaptan compared to placebo [11]. Subsequently, in 2014, Japan became the first country in the world to approve tolvaptan for the treatment of patients with ADPKD. Furthermore, the REPRISE trial (Replicating Evidence of Preserved Renal Function: An Investigation of Tolvaptan Safety and Efficacy) confirmed that tolvaptan was effective in patients with later stage ADPKD [12]. Tolvaptan was recommended as a grade 1A treatment in patients anticipated to progress rapidly in the practical guidelines for PKD in Japan [13]. Since 2014, a post-marketing surveillance (PMS), the SLOW-PKD surveillance (Samsca® Long-term surveillance of tolvaptan in PKD patients in real-world setting), has been conducted to evaluate the safety and efficacy of tolvaptan treatment in real-world clinical settings in Japan. This is the first report of the 5-year interim results of the SLOW-PKD surveillance.

## Materials and methods

### Surveillance design

This is a prospective, multicenter, observational, PMS 8-year surveillance. The surveillance was registered on ClinicalTrials.gov (NCT02847624). The target number of patients to be enrolled was 1600. The rationale behind the target number was to detect adverse drug reactions (ADRs) with an incidence of 0.3% or more, providing statistical power of 99%. In addition, assuming that the 4-year treatment continuation rate is 50%, we considered that it is possible to collect data on 800 patients which treated tolvaptan over 4 years.

In this report, we used the interim results (as of May 18, 2019) of the ongoing surveillance. This surveillance has been performed in compliance with Good Post-marketing Study Practice (GPSP).

### Surveillance population

Patients with a diagnosis of ADPKD, a TKV of more than 750 mL and an annual TKV slope of more than 5% as measured by magnetic resonance imaging (MRI), computed tomographic (CT) scanning, or ultrasound were included, provided they did not have serious renal impairment (estimated glomerular filtration rate [eGFR] of less than 15 mL/min/1.73 m^2^), liver injury, hypernatraemia, or difficulties with water intake, and were not pregnant. Informed consent and ethics committee approval are not required under the GPSP and were accordingly not mandatory in this surveillance.

### Data collection

The main data collected were as follows: demographic characteristics before tolvaptan treatment, TKV, kidney function (serum creatinine, eGFR), laboratory values (especially those related to liver function), adverse events (AEs), and CKD stage.

### Safety assessment

All events identified as AEs were aggregated, regardless of their causal relationship with tolvaptan therapy. The events for which a causal relationship to tolvaptan could not be ruled out by the attending physicians were categorized as ADRs. It was mandatory for liver function to be monitored at least every month. The incidence of AEs was summarized and AEs were coded using Medical Dictionary for Regulatory Activities (MedDRA) version 22.0.

Liver function was assessed based on alanine aminotransferase (ALT) levels. Abnormal levels were categorized based on the maximum ALT levels: > 30 and ≤ 90 IU/L, > 90 and ≤ 240 IU/L, and > 240 IU/L. Moreover, the number of patients who experienced abnormally high ALT, which is defined as more than 30 IU/L, were summarized. The ALT upper limit of normal (ULN) was set at more than 30 IU/L in accordance with the insurance guidance program issued by Japan’s Ministry of Health, Labor and Welfare in 2018 [14].

### Efficacy assessment

For the efficacy analysis, TKV was calculated by each attending physician based on MRI or CT; annual slopes were analyzed from the data of 253 patients with one pre-baseline and one post-baseline measurement included in the analysis.

The eGFR was measured at each investigative site, and the data from 416 patients were used for the analysis. At least one pre-baseline and one post-baseline (1 month or more after starting tolvaptan treatment) measurement from each patient were used for estimating slopes. Data collected within 1 month after start of treatment were excluded from the analysis since the hemodynamic effect was reported after 3 weeks in patients with ADPKD [15]. CKD stage G5 were excluded from the efficacy assessment in terms of non-approved indication.

### Statistical analysis

In efficacy analyses of TKV and eGFR, a mixed model with fixed effects of treatment, time and subject, interaction of treatment and time, interaction of subject and time as covariates, and random effects of intercept and time were used for patients with both pre-baseline and post-baseline treatment values. In the model, treatment was an indicator of pre-treatment or post-treatment observations, and time was the periods of days calculated from pre-baseline to baseline observations for the pre-treatment group, and from baseline to post-baseline observations for the post-treatment group. An unstructured variance–covariance matrix was assumed for the mixed model.

Observations of the eGFR were analyzed directly in the mixed model, however, log10 transformations of TKV were analyzed using a mixed model, and anti-log10, subtracted by 1 and then multiplied by 100, was applied to convert the estimates and their 95% CIs to a scale of percentage annual change in baseline TKV.

## Results

### Patient disposition and baseline characteristics

**Fig. 1:**
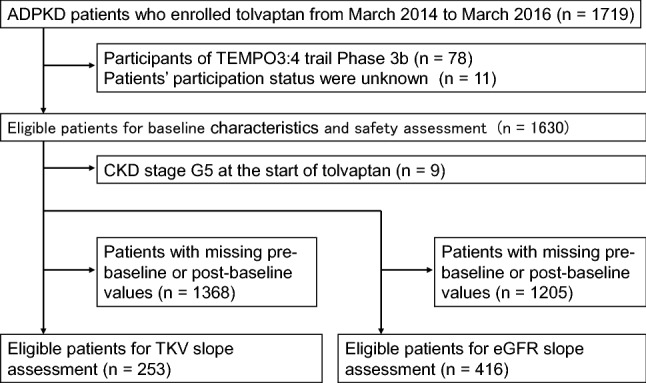
Flow chart of the surveillance population. ADPKD: autosomal dominant polycystic kidney disease

As of May 18, 2019, the data from a total of 1719 patients had been collected. All patients met the diagnostic criteria for ADPKD. A flow chart of the surveillance population is shown in Fig. 1. The baseline characteristics of 1630 patients treated with tolvaptan for the first time are reported; a further 78 patients were part of the phase 3b TEMPO extension study, the participation status of the remaining 11 patients in the TEMPO study is not known. In this report, we focus on the 1630 patients treated with tolvaptan for the first time. The patient demographics, complications and physical findings, and classifications are shown in Tables 1, 2 and 3, respectively. The baseline level of eGFR was 44.4 ± 21.7 mL/min/1.73 m^2^, TKV was 2158 ± 1346 mL, height adjusted TKV was 1301 ± 809 mL/m. Approximately half of the patients had CKD stage G3b (440 [27.0%]) or G4 (490 [30.1%]). Most of the patients belonged to Mayo class 1C (19.4%) or 1D (14.8%), but none to Mayo class 1A, while the data for 829 (50.9%) patients were missing because of missing body height and/or baseline TKV. Baseline characteristics of 253 patients eligible for TKV analysis on each CKD stage are reported in Table 4.

**Table 1:** Patient demographics

Variables, *n*	Mean ± SD
Age (years), *n* = 1630	49.7 ± 11.2
Height (cm), *n* = 1376	165.6 ± 9.1
Weight (kg), *n* = 1368	64.3 ± 12.8
Body Mass Index (kg/m^2^), *n* = 1336	23.3 ± 3.6
Blood urea nitrogen (mg/dL), *n* = 1588	23.9 ± 9.5
Serum creatinine (mg/dL), *n* = 1597	1.5 ± 0.7
eGFR(mL/min/1.73 m^2^), *n* = 1597	44.4 ± 21.7
Total kidney volume (mL), *n* = 965	2158 ± 1346
Height-adjusted total kidney volume (mL/m), *n* = 803	1301 ± 809

*eGFR* estimated glomerular filtration rate

All values are expressed as mean ± SD, and in the ratio of Male % value was provided.

**Table 2:** Complications and physical findings

Complications, *n* (%)	*n* = 1524
Hypertension	1380 (90.6)
Diabetes	61 (4.0)
Hyperlipidemia	395 (25.9)
Hyperuricaemia	628 (41.2)
Liver disease	716 (47.0)
Cystic liver	698 (45.8)
Other	46 (3.0)
Pancreatic cysts	17 (1.1)
Cerebral aneurysm	154 (10.1)
Kidney disease other than ADPKD	146 (9.6)
Urinary calculus	35 (2.3)
Cyst infection	24 (1.6)
Urinary tract infection	5 (0.3)
Other	663 (43.5)

Physical findings (symptoms), *n* (%)	*n* = 529
Low back pain/Flank pain (Including Nephralgia)	276 (52.2)
Feeling of abdominal distension	423 (80.0)
Anorexia	78 (14.7)
General malaise	95 (18.0)
Hematuria	128 (24.2)

**Table 3:** Classifications

Variables, *n* (%)	*n* = 1630
Gender, male	843 (51.7)
Female	787 (48.3)
Age group	
≤ 19	1 (0.1)
20–29	42 (2.6)
30–39	223 (13.7)
40–49	615 (37.7)
50–59	417 (25.6)
60–69	246 (15.1)
70–79	81 (5.0)
≥ 80	5 (0.3)
CKD stage	
G1	62 (3.8)
G2	299 (18.3)
G3a	328 (20.1)
G3b	440 (27.0)
G4	490 (30.1)
G5	9 (0.6)
Unknown	2 (0.1)
Total kidney volume	
< 750 mL	4 (0.2)
≥ 750– < 1500 mL	369 (22.6)
≥ 1500– < 3000 mL	403 (24.7)
≥ 3000– < 4500 mL	139 (8.5)
≥ 4500– < 6000 mL	31 (1.9)
≥ 6000 mL	19 (1.2)
Missing or before Day -90	665 (40.8)
Mayo classification	
1A	0 (0.0)
1B	152 (9.3)
1C	317 (19.4)
1D	242 (14.8)
1E	90 (5.5)
Missing	829 (50.9)

*CKD* chronic kidney disease, *SD* standard deviation

Baseline value is defined as the last available value on or prior to the first dose date

CKD stages were based on eGFR value (mL/min/1.73 m^2^): G1 (≥ 90), G2 (60 to 89), G3a (45 to 59), G3b (30 to 44), G4 (15 to 29), and G5 (< 15)

The baseline values for TKV and eGFR are defined as the last available values, on or prior to the first dose, extending back to Day -90

**Table 4:** Patient characteristic for patients in TKV analysis by CKD stage values

Patient characteristic	Entire population	CKD stage			
		G1 + G2	G3a	G3b	G4
Number of patients	*n* = 253	*n* = 64	*n* = 45	*n* = 66	*n* = 78
Age (years)	49.1 ± 11.4	40.2 ± 7.8	49.9 ± 10.9	50.0 ± 9.6	55.2 ± 11.2
Sex (Male; %)	49.4	48.4	46.7	51.5	50.0
Height (cm)	165.4 ± 9.3	165.3 ± 8.3	165.7 ± 10.5	165.5 ± 10.0	165.3 ± 9.1
Weight (kg)	64.3 ± 12.9	64.2 ± 13.3	63.6 ± 11.7	65.8 ± 14.6	63.8 ± 12.0
Body mass index (kg/m^2^)	23.4 ± 4.0	23.4 ± 4.0	22.9 ± 3.1	24.1 ± 5.1	23.2 ± 3.4
Systolic blood pressure (mmHg)	130.4 ± 15.2	127.1 ± 14.4	131.1 ± 17.5	134.9 ± 16.0	128.9 ± 12.6
Diastolic blood pressure (mmHg)	82.4 ± 11.8	80.8 ± 12.7	83.0 ± 10.7	85.2 ± 12.9	80.8 ± 10.1
Blood urea nitrogen (mg/dL)	23.0 ± 9.0	15.3 ± 4.1	18.6 ± 4.8	23.0 ± 5.1	31.8 ± 8.9
Serum creatinine level (mg/dL)	1.5 ± 0.7	0.8 ± 0.2	1.1 ± 0.2	1.5 ± 0.3	2.3 ± 0.6
e-GFR (mL/min/1.73 m^2^)	45.6 ± 22.5	77.6 ± 13.3	51.5 ± 3.9	37.1 ± 4.4	22.7 ± 4.4
Total kidney volume (mL)	2037 ± 1277	1361 ± 453	1856 ± 959	2556 ± 1851	2255 ± 1037
Height-adjusted total kidney volume (mL/m)	1223 ± 781	824 ± 268	1051 ± 501	1569 ± 1154	1375 ± 644
Total administration period (day)	835 ± 360	847 ± 345	922 ± 379	847 ± 353	763 ± 360
Starting dose (mg)	47.6	54.8	52.3	49.3	37.5
Average daily dose (mg/day)	66.0	75.7	74.3	72.8	47.5
Daily dose (most frequent dose) (mg/day)	67.6	76.2	74.0	75.9	50.0
Daily dose (final dose) (mg/day)	70.7	83.0	83.0	79.5	46.0

All values are expressed as mean ± SD, or in the case of sex as percentage of male subjects and dose as mean

Depending on patient characteristics, the patient number may differ because of missing data

### Treatment with tolvaptan

The starting dose of tolvaptan is summarized in Table 5. The higher the CKD stage, the lower the proportion of patients who were able to start at a dose of 60 mg/day. The mean tolvaptan daily dose was 63.0 ± 25.5 mg/day. The mean treatment period was 703 ± 417 days (median 731 days). During this period, 565 patients (34.7%) were discontinued from tolvaptan for the following reasons: adverse events (16.0%), change to another hospital (9.6%), other reasons (unspecified) (8.5%), lack of efficacy (1.7%), and patient did not visit (1.2%).

**Table 5:** Number of patients by starting dose and CKD stage (*N* = 1618)

	Starting dose	
CKD stage	< 60 mg	60 mg
G1 G2 G3a G3b G4 Total	12 (0.7%) 79 (4.9%) 95 (5.9%) 153 (9.5%) 286 (17.7%) 625 (38.6%)	50 (3.1%) 220 (13.6%) 232 (14.3%) 287 (17.7%) 204 (12.6%) 993 (61.4%)

Categorical variables are expressed as *n* (%)

### Safety

The ADRs with an incidence of 1% or more were listed in Table 6. These events were reported based on the physicians’ judgment that they were ADRs. In total, 668 (41.0%) of 1630 patients reported at least 1 ADR. The most frequent ADRs were hepatic function abnormal (8.3%), thirst (8.2%) and hyperuricaemia (6.9%) with an incidence exceeding 5%. Other ADRs were hypernatraemia (4.5%), liver disorder (3.1%), renal impairment (2.2%), pollakiuria (1.8%), nocturia (1.1%), blood creatinine increased (1.1%), dizziness (1.0%), nausea (1.0%), dehydration (1.0%), and insomnia (1.0%). Thirst, hypernatraemia, pollakiuria, and nocturia were anticipated to be common adverse events based on earlier studies of tolvaptan treatment. [16, 17] The notable ADRs were summarized in Table 7. Hepatic- and thirst-related ADRs were reported in 226 and 133 patients, respectively.

**Table 6:** Occurrence of adverse drug reactions (> 1.0%) after tolvaptan administration

Preferred terms, *n* (%)	Number of patients
Any ADR	668 (41.0)
Hepatic function abnormal	136 (8.3)
Thirst	133 (8.2)
Hyperuricaemia	113 (6.9)
Hypernatraemia	74 (4.5)
Liver disorder	50 (3.1)
Renal impairment	36 (2.2)
Pollakiuria	30 (1.8)
Nocturia	18 (1.1)
Blood creatinine increased	18 (1.1)
Dizziness	17 (1.0)
Nausea	16 (1.0)
Dehydration	16 (1.0)
Insomnia	16 (1.0)

Categorical variables are expressed as *n* (%)

**Table 7:** Occurrence of adverse drug reactions by safety specification category

Safety specifications	Number of patients
Preferred term	
Acute hepatic failure and hepatic function disorder	226
Acute hepatic failure	1
Alanine aminotransferase abnormal	1
Alanine aminotransferase increased	9
Aspartate aminotransferase abnormal	1
Aspartate aminotransferase increased	11
Gamma-glutamyltransferase increased	15
Hepatic function abnormal	136
Hepatic steatosis	1
Hepatitis acute	1
Hyperbilirubinaemia	2
Liver disorder	50
Liver function test abnormal	2
Transaminases increased	1
Hepatic enzyme increased	11
Drug-induced liver injury	5
Hepatic cancer	1
Liver function test increased	3
Thirst	133
Lip dry	1
Thirst	133
Hypernatraemia	81
Blood sodium increased	7
Hypernatraemia	74
Renal failure and impairment	80
Azotaemia	0
Blood creatinine increased	18
Blood urea increased	14
Glomerular filtration rate decreased	2
Renal disorder	2
Renal failure	11
Protein urine present	1
Postrenal failure	1
Renal impairment	36
Chronic kidney disease	3
Acute kidney injury	3
Prerenal failure	2
End stage renal disease	1
Hyperkalaemia	23
Blood potassium increased	4
Hyperkalaemia	13
Muscle spasms	6
Dehydration	17
Dehydration	16
Weight decreased	2
Thrombosis and thromboembolism	7
Cerebellar infarction	1
Cerebral infarction	2
Disseminated intravascular coagulation	1
Pulmonary embolism	2
Retinal artery occlusion	1

In total, 11 deaths occurred during this reporting period and, of those, 2 were considered as having a causal relationship to treatment with tolvaptan. One death was in a 63-year-old female patient who was found to have a liver tumor after 7 months. She underwent surgery but the tumor could not be removed and she eventually died. Another 51-year-old female patient developed a cyst infection after 5 months. She subsequently developed disseminated intravascular coagulation and liver/renal impairment.

The details of hepatic dysfunction are described below. An ALT increase to over 30 IU/L was observed in 483 of 1468 patients (32.9%) in Table 8. Of these, 361 patients (24.6%) had ALT increased to between 30 and 90 IU/L, 69 patients (4.7%) had ALT increased to between 90 and 240 IU/L, and 53 patients (3.6%) had ALT increased to over 240 IU/L which is 8 times the ULN according to the US Food and Drug Administration (FDA) Guidance for Industry: Drug-induced Liver Injury, 2009 [18].

**Table 8:** Patients with increased ALT with baseline ALT 30 IU/L or below by maximum ALT level (*N* = 1468)

ALT level, *n* (%)	Number of patients with increased ALT
Total	483 (32.9%)
> 30 and ≤ 90	361 (24.6%)
> 90 and ≤ 240	69 (4.7%)
> 240	53 (3.6%)

Categorical variables are expressed as *n* (%)

## Efficacy

### Assessment of TKV slope

**Fig. 2:**
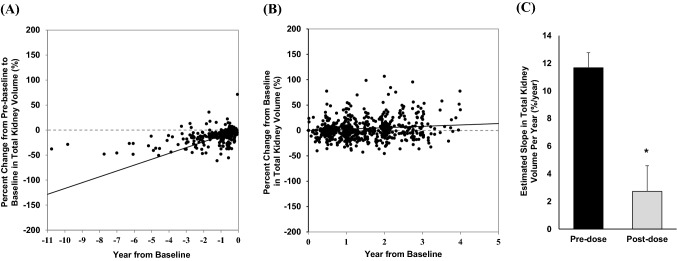
Effect of tolvaptan on TKV percentage change. The estimated slope is based on a mixed effect model with fixed factors of pre/post group, time, and subject; pre/post group time interaction and subject time interaction as covariates. (**A**) Scatter plot and estimated slope before tolvaptan administration, (**B**) Scatter plot and estimated slope after tolvaptan administration, (**C**) Comparison of the estimated percentage change in the TKV slope between pre (black) and post (grey) tolvaptan initiation (*P* < 0.0001)

According to the Japanese product information, tolvaptan is contraindicated in patients with an eGFR of less than 15 mL/min/1.73 m^2^. Therefore, out of 1630 patients, 9 patients were excluded because of CKD stage G5, leaving 1621 patients to whom the drug was administered in accordance with the instructions on proper use. The TKV analysis was performed in 253 patients with both pre-baseline and post-baseline on-treatment TKV values. Prior to tolvaptan treatment, the estimated percent change from pre-baseline to baseline in TKV was 11.68%/year (Fig. 2A). The estimated percent change from baseline to post-baseline in TKV was 2.73%/year, indicating that the kidney growth rate was lower after tolvaptan treatment (Fig. 2B). The change was statistically significant with a treatment comparison p value of < 0.0001 (Fig. 2C).

### Assessment of eGFR slope

**Fig. 3:**
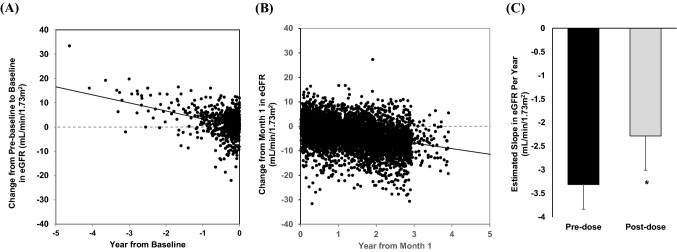
Effect of tolvaptan on eGFR delta value change. The estimated slope is based on a mixed effect model with fixed factors of pre/post group, time, and subject; pre/post group time interaction and subject time interaction as covariates. (**A**) Scatter plot and estimated slope before tolvaptan administration, (**B**) Scatter plot and estimated slope after tolvaptan administration, (**C**) eGFR estimated slope delta value change comparison pre (white) and post (grey) tolvaptan initiation (*P* = 0.0403)

Analysis of estimated slope in eGFR before treatment and after 1 month of treatment was performed for 416 patients. The estimated slope was − 3.31 ± 0.53 mL/min/1.73 m^2^/year in the pre-treatment period (Fig. 3A) and − 2.28 ± 0.73 mL/min/1.73 m^2^/year in the period after 1 month of treatment (Fig. 3B), a difference that was statistically significant (*p* = 0.0403) (Fig. 3C).

## Discussion

In the present surveillance, we analyzed the safety and efficacy of tolvaptan in real-world settings based on the PMS database containing data from March 2014 to May 2019. We compared the patient baseline characteristics of this surveillance with TEMPO 3:4 and REPRISE. It was speculated that the baseline TKV and eGFR were worse than those observed in TEMPO 3:4 because of the exclusion of patients with a creatinine clearance of less than 60 mL/min from the TEMPO 3:4 study. In REPRISE, the baseline eGFR was similar to this surveillance.

The overall safety profile of tolvaptan was consistent with that seen in previous studies [11, 12]. In this surveillance, the percentage of aquaretic AEs (AAEs) such as thirst, pollakiuria, nocturia, and dehydration was lower than in the REPRISE trial. This can be attributed to patients probably maintaining sufficient water intake during tolvaptan treatment in accordance with the physician’s instructions.

ADRs regarding hepatic function were observed in 136 patients as hepatic function abnormal and in 50 patients as liver disorder in Table 6. Based on the liver function test results, an ALT increase of > 30 IU/L and > 90 IU/L were observed in 32.9% and 8.3% of all patients (Table 8), respectively. In the REPRISE study, the elevation of ALT values to more than three times the ULN, was observed in 5.6% of patients in tolvaptan group [12]. It seemed that the incidence of ALT elevation over 90 IU/L in this surveillance was higher than that of the REPRISE study. Considering the study period on both studies, this surveillance was about twice as long as REPRISE. Therefore, the ALT elevation ratio may be expected to be larger in this surveillance. However, it is difficult to compare because the methods and reference ranges are not the same in both studies. We will accumulate more data for further analysis. Watkins et al. reported that the onset of liver injury was observed between 3 and 18 months after initiation of tolvaptan treatment by analyzing the database of clinical trials [19]. It was suggested that the importance of periodic monitoring of hepatic function was confirmed to detect the abnormality early.

The efficacy and safety of tolvaptan in patients with ADPKD was confirmed in two studies, TEMPO 3:4 [11] and REPRISE. [12] TEMPO 3:4 was the pivotal study and demonstrated the efficacy and safety of tolvaptan over a 3-year treatment period. However, it had certain limitations due to the inclusion criteria, age and disease stage. On the other hand, the REPRISE study focused on later stage ADPKD patients, i.e., including stage G4. The primary outcome of the REPRISE study was the demonstration of tolvaptan efficacy in terms of the change in eGFR from baseline to follow-up. However, TKV change was not analyzed. Real-world data are therefore important to understand the risk/benefit profile of tolvaptan.

The growth rate of TKV observed before tolvaptan treatment was 11.68%/year, whereas it was 5.51%/year in TEMPO 3:4 and 5.0%/year in the Japanese subgroup of the placebo group [11, 20]. This result indicates a TKV growth rate of more than double that observed in TEMPO 3:4. Pre-dose values in this study may be overestimated due to the high variability in the timing of measurements, which may affect the value of the annual rate of change. Comparisons with the TEMPO trial, which was prospectively observed for 3 years, are not considered appropriate. A potential explanation for the difference may be the difference in populations. Patient characteristics indicate that patients in the current surveillance have more advanced ADPKD. The annual rate of TKV increase was reported as between 3.7% and 4.7% depending on the group, in patients with TKV under 750 mL at baseline in the CRISP study [21]. Yu et al. observed that patients with a larger TKV had a faster rate of eGFR decline, thus correlating TKV at baseline with the worsening of CKD stages [22]. In the post hoc analysis in the HALT-PKD study, non-progressors had a significantly smaller TKV, and the mean slope for TKV increase was 4.8% for non-progressors in contrast to 6.4% for linear progressors [23], indicating a variation in TKV progression depending on the baseline values. If the annual change in TKV depends on the baseline values, a faster TKV growth rate could be expected in the current surveillance.

Therefore, further evaluation of patient baseline characteristics is warranted to assess efficacy in stage G4 patients to understand tolvaptan effect on progressive renal interstitial fiblosis [24]. Moreover, the dose of tolvaptan should be regarded as a key factor. The average dose used in TEMPO 3:4 was 95 mg in 88% of patients in the tolvaptan group [11]. In REPRISE, 82.3% of patients were treated with a dose of 120 mg/day [12]. When compared to the randomized clinical trials, much lower doses were used to treat stage G4 patients in the current surveillance (Table 4). Sub-analysis by CKD stages and Mayo classification demonstrated a statistically significant clinical effect between pre- and post-tolvaptan dosing from stage G1 to G3b in CKD stage and from Class 1B to 1D in Mayo classification, but no significant efficacy was observed G4 in CKD and 1E in Mayo classification patients (Supplemental Fig. 1 and Table 1). These results suggest that tolvaptan treatment in stage G4 and Class 1E patients with already impaired renal function was less effective, although further investigation is required. Therefore, it is important to start tolvaptan treatment before their renal function deteriorates further.

Limitations of this surveillance are as follows: all data in this interim analysis were collected during the first 5 years after approval and the average treatment period was 703 days. The discontinuation rate of patients due to AEs could not be estimated accurately as tolvaptan treatment was reinitiated in some patients. The efficacy assessments for eGFR and TKV slope were performed in a small number of patients. All data came from daily clinical practice, so that laboratory data might not be measured under the same conditions. The measurement methods of TKV were not standardized. TKV were measured mostly by CT or MRI but some by ultrasound.

In conclusion, the safety and efficacy of tolvaptan in patients with ADPKD were assessed in the real-world condition. The comparable safety and efficacy for TKV and eGFR was observed in relation to the previous pivotal two randomized control trials. In particular, it was speculated that the frequency of liver injury was in the same ballpark with the REPRISR study. For detecting the hepatic impairment earlier, it was considered important to monitor the hepatic function during treatment with tolvaptan for ADPKD.

## Supplementary Information

Below is the link to the electronic supplementary material.

CKD and Mayo classification analysis of TKV progression pre- and post- tolvaptan treatment. The comparison of delta TKV value change between pre (black) and post (grey) tolvaptan administration by CKD stage and by Mayo classification (PDF 94 KB)

Supplementary file2 (DOCX 18 KB)
