# Supplementary figures and images for: Safety and efficacy of Tolvaptan in real-world patients with autosomal dominant polycystic kidney disease- interim results of SLOW-PKD surveillance
Source: Clin Exp Nephrol. 2021 Jul 6;25(11):1231–9. doi: 10.1007/s10157-021-02100-0 (PMC8460520; doi:10.1007/s10157-021-02100-0)

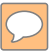**(A)**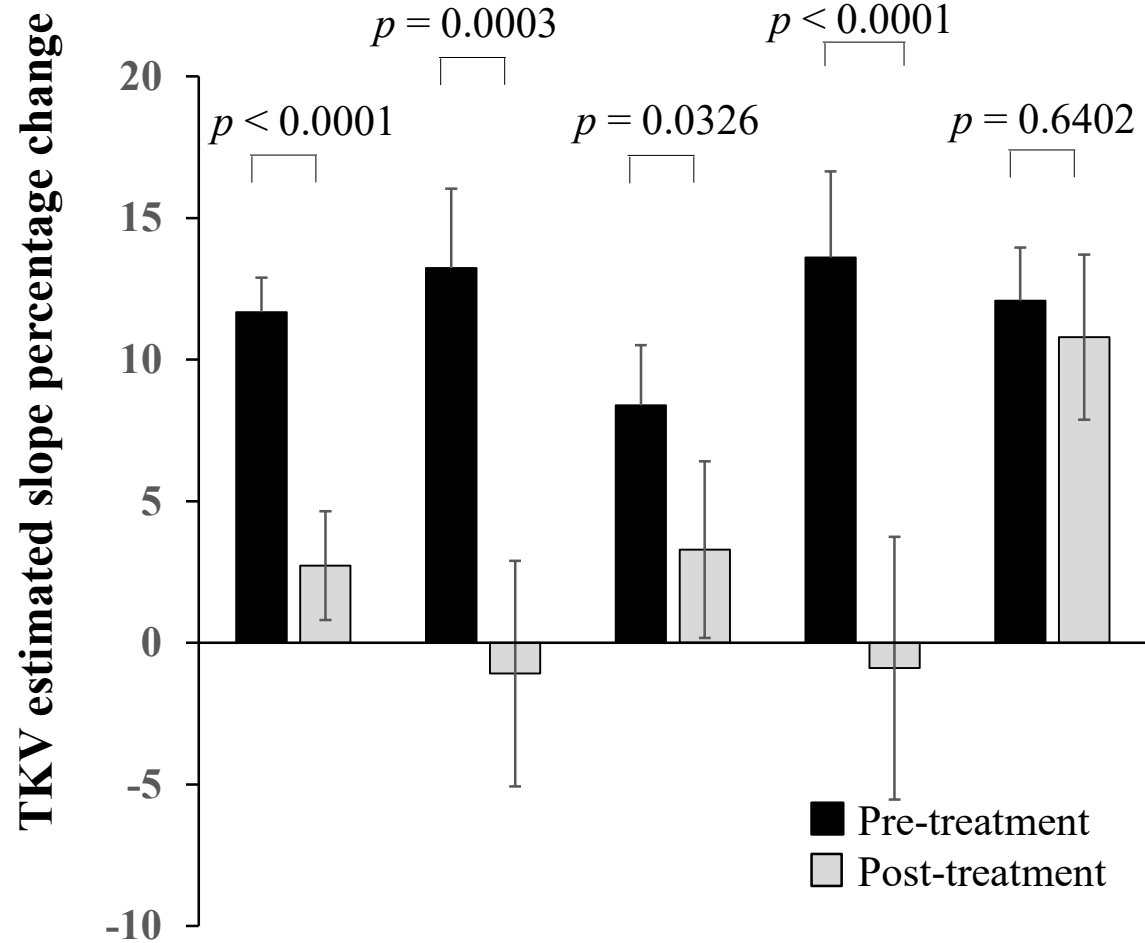**(B)**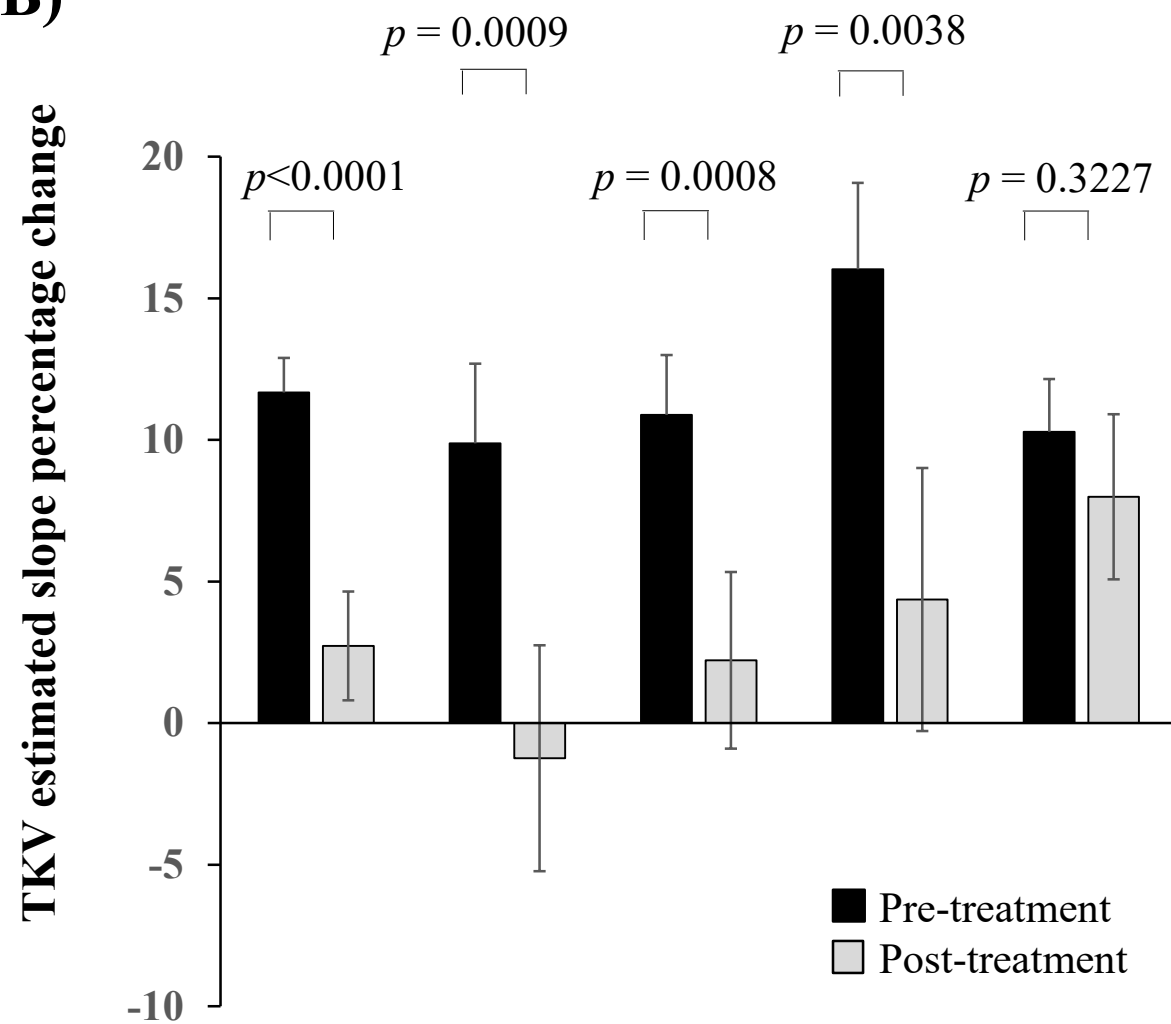

Supplement: Supplementary file 1 — CKD and Mayo classification analysis of TKV progression pre- and post- tolvaptan treatment. The comparison of delta TKV value change between pre (black) and post (grey) tolvaptan administration by CKD stage and by Mayo classification (PDF 94 KB) [file 10157_2021_2100_MOESM1_ESM.pdf]
