# Supplementary material for: Safety and efficacy of Tolvaptan in real-world patients with autosomal dominant polycystic kidney disease- interim results of SLOW-PKD surveillance
Source: Clin Exp Nephrol. 2021 Jul 6;25(11):1231–9. doi: 10.1007/s10157-021-02100-0 (PMC8460520; doi:10.1007/s10157-021-02100-0)
Supplement: Supplementary file 2 — Supplementary file2 (DOCX 18 KB) [file 10157_2021_2100_MOESM2_ESM.docx]

**Supplemental Table 1. Subgroup analysis by patient characteristic for Mayo classification**

| **Patient characteristic** | **Entire** | **Mayo classification** | | | |
| --- | --- | --- | --- | --- | --- |
|  | **population** | **1B** | **1C** | **1D** | **1E** |
| Number of patients | n=225 | n = 43 | n = 92 | n = 63 | n = 27 |
| Age (years) | 49.1 ± 11.5 | 58.6 ± 9.0 | 51.0 ± 11.5 | 44.6 ± 7.7 | 37.8 ± 7.6 |
| Sex (Male; %) | 49.3 | 27.9 | 40.2 | 65.1 | 77.8 |
| Height (cm) | 165.4 ± 9.3 | 161.5 ± 9.4 | 163.5 ± 8.4 | 169.0 ± 8.6 | 169.8 ± 9.8 |
| Weight (kg) | 64.3 ± 12.8 | 56.8 ± 9.5 | 62.2 ± 11.5 | 67.9 ± 11.2 | 76.3 ± 16.0 |
| Body Mass Index (kg/m^2^) | 23.4 ± 4.0 | 21.7 ± 3.6 | 23.1 ± 3.3 | 23.7 ± 3.4 | 26.8 ± 6.2 |
| Systolic blood pressure (mmHg) | 130.4 ± 15.3 | 129.4 ± 18.1 | 129.9 ± 15.0 | 131.6 ± 14.5 | 131.0 ± 13.7 |
| Diastolic blood pressure (mmHg) | 82.2 ± 11.8 | 77.0 ± 12.2 | 81.1 ± 10.6 | 85.5 ± 11.6 | 87.4 ± 11.7 |
| Blood urea nitrogen (mg/dL) | 22.7 ± 8.8 | 24.3 ± 11.1 | 22.1 ± 9.1 | 21.9 ± 6.7 | 24.2 ± 7.9 |
| Serum creatinine level (mg/dL) | 1.5 ± 0.7 | 1.4 ± 0.6 | 1.4 ± 0.7 | 1.5 ± 0.7 | 1.7 ± 0.7 |
| e-GFR (mL/min/1.73m^2^) | 45.9 ± 21.9 | 41.5 ± 19.6 | 46.9 ± 23.2 | 47.6 ± 21.8 | 44.9 ± 21.2 |
| Total kidney volume (mL) | 2030 ± 1323 | 1049 ± 265 | 1684 ± 782 | 2520 ± 1470 | 3628 ± 1558 |
| Height-adjusted total kidney volume (mL/m) | 1223 ± 781 | 652 ± 174 | 1036 ± 497 | 1494 ± 880 | 2131 ± 894 |
| Total administration period (day) | 838 ± 355 | 856 ± 325 | 902 ± 366 | 763 ± 359 | 768 ± 322 |
| Starting dose (mg/day) | 47.3 | 43.8 | 47.9 | 48.8 | 46.9 |
| Average daily dose (mg/day) | 66.1 | 63.3 | 65.8 | 68.3 | 66.1 |
| Daily dose (most frequent dose) (mg/day) | 67.5 | 65.1 | 66.4 | 70.4 | 68.9 |
| Daily dose (final dose) (mg/day) | 71.2 | 71.0 | 67.7 | 74.5 | 75.6 |

All values are expressed as mean ± SD, or in the case of sex as percentage of male subjects and dose as mean.

Patients classified as Mayo stage G5 are not included. Depending on patient characteristics, the patient number may differ because of missing data.
